# An optimal method for diagnosing heart disease using combination of grasshopper evalutionary algorithm and support vector machines

**DOI:** 10.1016/j.heliyon.2024.e30363

**Published:** 2024-04-25

**Authors:** Wei Zhou, Hongbo Liu, Rui Zhou, Jiafu Li, Sina Ahmadi

**Affiliations:** a Southwest Medical University, Clinical Medicine School, Luzhou, 646000, Sichuan, China; b People's Hospital of Leshan, Department of Cardiology, Leshan, 614000, Sichuan, China; c The Affiliated Hospital of Southwest Medical University, Department of Cardiology, Luzhou, 646000, Sichuan, China; d Master of Science of Information Technology Engineering, Department of Computer Engineering, West Tehran Branch, Islamic Azad University, Tehran, Iran

**Keywords:** Heart disease, Data mining, Support vector machine, Locust evolutionary algorithm, Performance criteria

## Abstract

Due to the importance of accurate diagnosis and prompt treatment of this condition, the medical world is searching for a solution for its early detection and efficient treatment. Heart disease is one of the leading causes of death in modern society. With the development of computer science today, this issue can be resolved using computers. Data mining is one of the solutions for diagnosing this illness. One of the cutting-edge disciplines, data mining, can aid in better decision-making in many areas of medicine, including disease diagnosis and treatment. In order to improve diagnosis accuracy, a combination method using the evolutionary algorithms locust and support vector machine has been tested in this study. Use should be made of heart disease. Because of the hybrid nature of this approach, normalization is actually carried out in three steps: first, by using pre-processing operations to remove unknown and outlier data from the data set; second, by using the locust evolutionary algorithm to choose the best features from the available features; and third, by classifying the data set using a support vector machine. The accuracy criterion for the proposed method compared to Niobizin methods, neural networks, and J48 trees improved by 18 %, 30 %, and 24 %, respectively, after implementing it on the data set and comparing it with other algorithms used in the field of heart disease diagnosis.

## 1 Introduction

Technological improvement in computers and other sciences has led to amazing advances in the gathering and storage of data of various forms [1,2]. For the purpose of keeping data about business transactions, agriculture, traffic, the Internet, astronomy, phone calls, and other subjects, large databases are required [[3], [4], [5]]. There have been attempts since the late 1980s to retrieve the data hidden in this massive volume of data that can't be retrieved with the traditional techniques for accessing and retrieving data from databases [6]. The intense competition in scientific, social, political, and military fields has made it necessary to design systems that can, on the one hand, quickly find the information users need with little human intervention and, on the other hand, find appropriate analysis methods [[7], [8], [9]]. Conversely, a large amount of data was utilized to sense it [10,11]. The process of extracting information from databases from a new angle is known as data mining, and it was first used in the early 1990s [12]. As of right now, data mining is the most important method for effectively utilizing large amounts of data, and its importance is only increasing. Numerous fields and occupations, such as commerce, medicine, engineering, computer science, industry, quality assurance, communication, and agriculture, can make use of it [13,14].

Cardiac disease is a condition that alters heart function [15]. A variety of factors, including smoking, high blood pressure, cholesterol, obesity, inactivity, and a family history of heart disease, are heart disease risk factors. Prevention is the best way to lower the risk of heart disease [[16], [17], [18]]. The physical examination, signs, and symptoms of the patient typically inform the diagnosis. Nearly all physicians utilize knowledge and experience to foresee cardiac illness. In the field of medicine, figuring out the cause of an illness is difficult and time-consuming [19,20]. Heart disease prediction is a complicated subject that could result in erroneous assumptions and undesired outcomes. Therefore, diagnosing this illness without invasive procedures is crucial [[21], [22], [23]]. Cardiac patient tests and examinations have collected numerous data points, which traditional methods would find costly to process [24]. If there were fewer of these factors influencing the diseases, humans could review the data and identify them; however, due to the large number of factors, data mining techniques are needed to analyze the data [[25], [26], [27]]. We have identified the factors that have the greatest impact on this condition [28]. The methods for forecasting heart illness will be vital and given top priority in the investigations in order to aid in the early diagnosis of heart problems, which will help to prevent bad effects and extravagant expenses through the knowledge of data mining and enhance people's lives [29,30].

The support vector machine has drawn a lot of interest in recent research, and the majority of studies use this sort of machine to speed up system responsiveness. Support vector machines will be employed in the proposed study to classify the data set. The data set is initially extracted from the UCI website as part of the work procedure. Age, gender, blood sugar, cholesterol, and other factors are among the 14 characteristics included in this data collection for the diagnosis of heart disease. The best subset of features will be chosen at the start of the suggested technique, utilizing the evolutionary algorithm, in order to carry out the operation quickly. In reality, the grasshopper evolutionary algorithm will be used to choose the features that have the most influence on the diagnosis of heart disease. To accomplish this, a set of features will be chosen at random, and for this set, the fitness level, a measure of the amount of inaccuracy brought on by the categorization of the data set using the present features, will be computed. The feature set with the lowest fitness value will be chosen as the best feature subset once the algorithm's iteration stages are complete. The data set will next be classified using the support vector machine algorithm based on the characteristics chosen by the evolutionary algorithm, and at the conclusion of the job, we will determine the proposed method's efficiency criteria. The proposed method is anticipated to be more accurate than existing algorithms currently used in the field of heart disease prediction, per the study process. Following is a summary of the authors' contributions to this study.

• Choosing the best subset of features using the new evolutionary algorithm will speed up the suggested technique.
• Classifying the data set using the support vector machine technique in order to identify heart disease.

The continuation of the article is sub-organized. The review of previous studies is described in section 2. The diagnosis of heart disease using the proposed hybrid approach is explained in detail in Section 3. In section 4, the evaluation and implementation of the proposed method is given. Section 5 states the conclusion of the research, and finally, in Section 6, the future works are stated.

## 2 Related works

By combining decision tree, neo-business, and neural network data mining techniques, researchers have created an intelligent system for predicting cardiac disease. Whereas conventional decision support systems fall short, IHDPS is able to respond to complex "what" inquiries. You may predict your risk of heart disease using medical characteristics like age, gender, blood pressure, and blood sugar. IHDPS, which utilizes the.NET framework, is web-based, user-friendly, scalable, dependable, and upgradeable [31]. In Ref. [32], a system is created to show how artificial neural networks can be used to diagnose neonatal diseases and predict diseases. The suggested method uses model BP learning algorithm and multi-layer propteron training to diagnose and forecast newborn illnesses. The ANN design was trained using the feedback technique, which has also been evaluated for several types of infant illnesses. About 94 parameters of symptoms and indicators have been examined in this model. This study demonstrates that ANN is considerably more stable and accurate at predicting baby heart illness than prior studies, which had an accuracy of 0.75 percent. Researchers examined decision trees, artificial neural networks, machine learning, and RIPPER classification for data on cardiovascular illness in Ref. [33]. The sensitivity, specificity, accuracy, error rate, true positive rate, and false positive rate are used to compare the effectiveness of these procedures. The 10-fold symmetric validation approach was employed to measure the indirect estimation of these prediction models. The RIPPER, decision tree, ANN, and SVM errors are 2.756, 0.2755, 0.2248, and 0.15888, respectively, same like in our results. ANN, SVM, RIPPER, and decision trees are each 81.08 %, 79.05 %, 06.08 %, and 84.12 % accurate, respectively [34]. The analysis demonstrates that SVM, out of these four classification models, accurately and with the least amount of error predicts cardiovascular illness.

Researchers have suggested a decision support method for identifying congenital cardiac disorders in another study. The recurrent neural network is implemented in the suggested system using the GUI feature of MATLAB. A multi-layer feedforward neural network with supervised delta learning was utilized to train the recurrent neural network in this study. The signs, symptoms, and findings of a patient's physical examination served as the study's source of data. The proposed system had an accuracy rate of 90 % [35]. A neural network-based approach for predicting cardiac illnesses, high blood pressure, and diabetes was developed in Ref. [36]. For training and testing, a set of 78 records with 13 features is employed. He suggested a supervised network for the diagnosis of heart disease and trained it with a backward method. The algorithm creates a list of potential diseases that the patient might have based on the unknown data entered by the doctor and the unknown data it discovers from the training data. In order to prevent heart attacks, a specialized strategy for finding important patterns from heart disease collections is provided in Ref. [37]. The data warehouse must first go through preprocessing in order to be ready for mining. The K-Means clustering approach is used to cluster the heart disease repository after it has undergone preprocessing. The MAFIA algorithm is then used to extract common patterns for heart disease from the retrieved data. Furthermore, patterns that are essential for preventing heart attacks are chosen based on a considerable, determined weight. To accurately predict heart attacks, the neural network is trained using a few key significant patterns [38]. proposes an effective method for probabilistic neural network (PNN)-based smart heart disease prediction. First, the Cleveland Heart Disease Database provided a dataset with 13 medical variables. The k-means clustering algorithm is used to cluster this data collection. Using the chosen data set, the PNN with radial basis function is trained. The chosen significant patterns are trained using the current neural network-based method, which combines a back-and-forth algorithm (BP) with a multi-layer preprocessing (MLP) algorithm [39]. The multi-layer feedforward network of perceptron neural networks is one sort of neural mechanism. Table 1 compares these approaches in light of the justifications provided for each of the prior studies done in the area of heart disease prediction.

**Table 1:** Review of existing works based on different parameters.

Ref.	Purpose	Method	Parameters	Advantages	Disadvantages
[19]	Cardiovascular disease prognosis	Incorporating techniques from both machine learning and data mining	precision	Coordinate data from multiple sources and spot trends quickly.	Challenges in data interpretation and heightened susceptibility to bias
[20]	Cardiovascular disease prognosis	Chi-square and principal component analysis for cardiac disease diagnosis	precision	The method's simplicity and ease of use	It does poorly when used on a collection of features that are dependent on one another.
[40]	Cardiovascular disease prognosis	Heart disease can be identified using a variety of classification techniques and a set of ideal criteria.	precision	- Quick and simple classification. - It also functions well when there are more than two categories.	For dependent and non-independent data, it performs poorly.
[41]	Diabetes, cancer, and heart disease are forecast as three diseases.	Algorithm for Simple Bayes	Using K-MEANS to cluster	1 Both prescriptions and doctor's prescriptions are part of the planned system. 2. Every patient sees a different specialist.	1. The significance of selecting the clustering's number of clusters. 2 The outcomes depend on the specialists' assessments of the organization.
[27]	clever heart disease forecasting	Making use of probabilistic neural networks	To assess the aforementioned algorithm, use the ROKCH methodology.	In order to improve the suggested system's accuracy, neural networks are used.	Using a neural network technique requires training time.
[42]	extracting heart disease pattern warehouses to stop heart disease	Using MAFIA pattern extraction and K-Means clustering	helpful patterns for preventing heart disease	the potential for analyzing the condition using the model.	the time required for pre-processing before applying the algorithm to predict heart disease.
[43]	Congenital heart disease diagnosis	Recurrent neural network usage	accuracy, mistake	boosting accuracy with neural networks.	solely using delta law to constrain network training.
[44]	Neonatal heart disease forecasting	By means of artificial neural networks	94 characteristics are used to diagnose the illness.	- Increased stability. - Improving precision	neural network layer training takes a long time.

## 3 Proposed method

Support vector machines and grasshopper evolutionary algorithms are used to develop the suggested technique. We will first construct a support vector machine using the heart disease input parameters, and its output will indicate whether heart disease is present or not [45]. Support vector machines can detect issues in a variety of ways. For issues involving features, the diagnosis model is typically in the general form (1):

*X*_*i*_*=f(x*_*1*_*….x*_*n*_*)* (1)

The detection value obtained by applying the function f to the characteristics *x*_*1*_ to *x*_*n*_ is represented by the variable *X* in this way. Support vector machines can be trained to predict one or more independent variables, but the network's proper training is essential for accurate diagnosis. The speed of heart disease diagnosis by the support vector machine can be enhanced to an acceptable degree if the amount of features input to the machine can be decreased [46,47]. The dimensions in this study are reduced using the grasshopper evolutionary method.

**Fig. 1:**
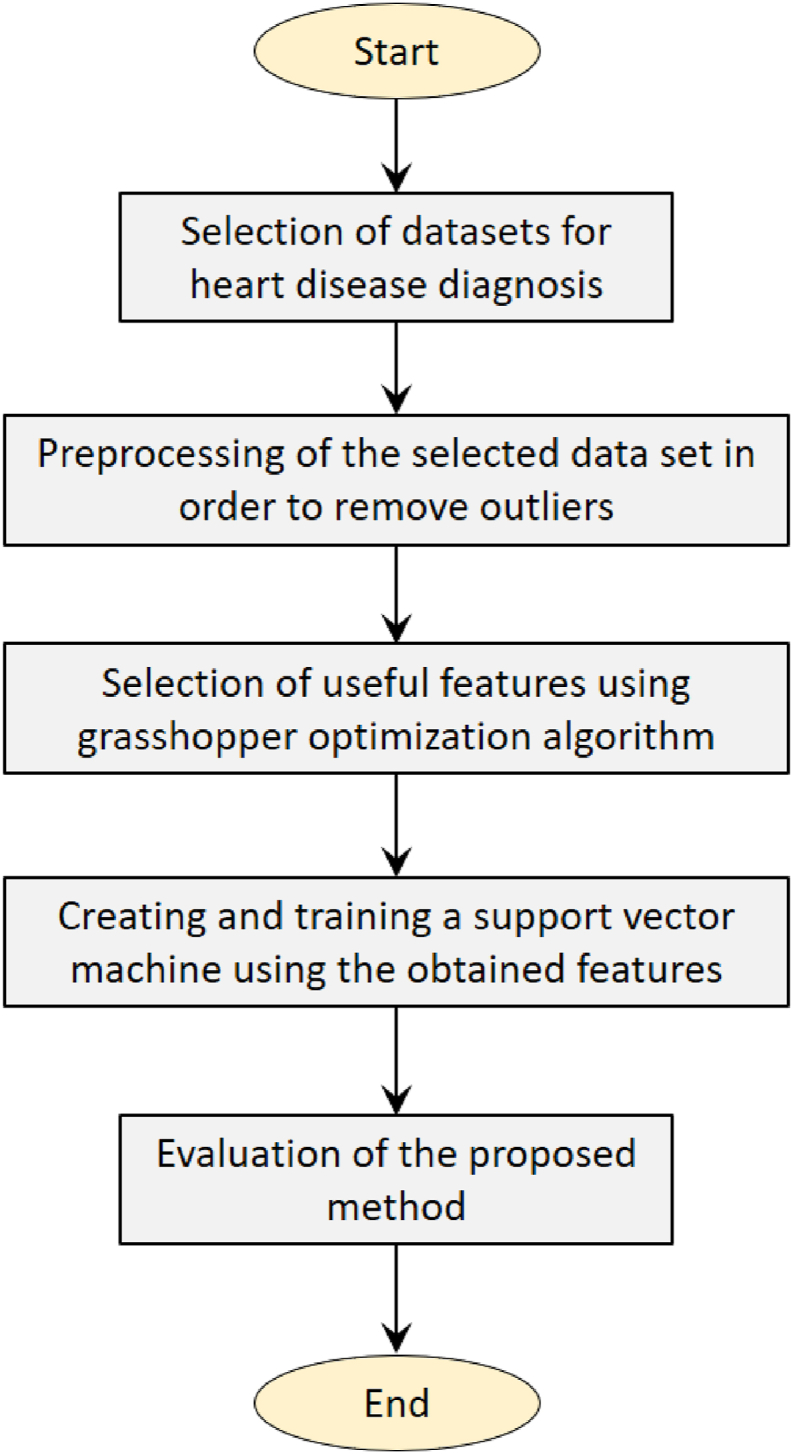
Process of the proposed method.

The five stages of the suggested methodology are, in order, the following: choosing a data set, preprocessing the data, choosing closed-type features, creating a support vector machine using the features collected, and training the support vector machine. The proposed method's flowchart is shown in Fig. 1.

### 3.1 Data set selection

The Heart Disease Database, a common data collection from the UCI website, has been utilized to apply the suggested strategy. The 14 features in this dataset are listed in Table 2.

**Table 2:** Description of data set characteristics.

Feature	parameter	How to describe	according
Age	Age	Numerical	Year
Sex	gender	nominal	Two cases, male and female
Cp	Type of chest pain	nominal	A number between 1 and 4
Trestbps	resting blood pressure	Numerical	Hmm hg
Chol	Cholesterol	Numerical	Mg/dl
Fbs	blood sugar	Numerical	Mg/dl
Restecg	Electrocardiography	Numerical	A number between 0 and 2
Thalach	Maximum heart rate	Numerical	Between 1 and 300
Exang	Exercise induced angina	Numerical	A number between 0 and 1
Oldpeak	Exercise-induced depression	Numerical	–
Slope	The slope of the peak part of the exercise	Numerical	A number between 1 and 3
Ca	Number of main vessels	Numerical	A number between 0 and 3
Thal	Heart failure condition	Numerical	A number between 3, 6 and 7
Num	Determining the status of heart disease	Numerical	A number between 0 and 1

Table 2 makes it evident that there are 13 features for predicting heart disease, making a total of 14 features when the column for predicting heart disease is included. The data set and normalization are finished. The issues with characteristics that have no value are resolved by the preprocessing procedure. to produce an entirely balanced data set for carrying out procedures.

### 3.2 Data preprocessing

There might not be any data values in some rows of the chosen attributes in the specified data collection. There are numerous techniques for preparing dataset records; Table 3 lists these techniques for each attribute.

**Table 3:** Removing any variables' missing values from the dataset.

Variable	How to remove missing values	Variable	How to remove missing values
**Age**	Average of similar records	**Sex**	most likely value among similar records
**CP**	most likely value among similar records	**Trestbps**	Average of similar records
**Chol**	Average of similar records	**Fbs**	Average of similar records
**Restecg**	most likely value among similar records	**Thalach**	Average of similar records
**Exang**	most likely value among similar records	**OldPeak**	Average of similar records
**Slope**	most likely value among similar records	**Ca**	most likely value among similar records
**Thal**	most likely value among similar records		

### 3.3 Choosing certain data set attributes

**Fig. 2:**
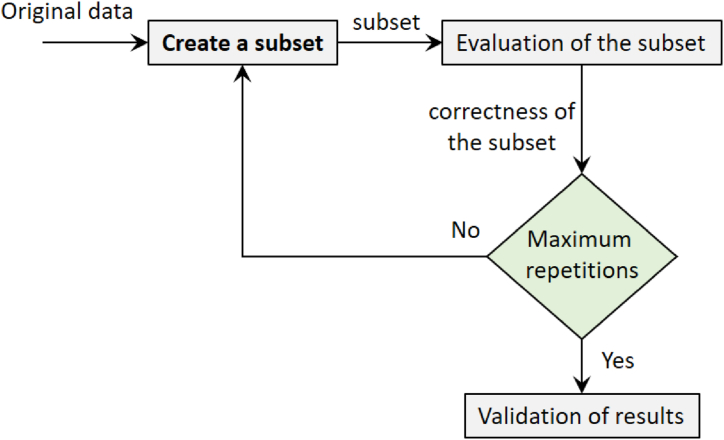
Feature selection process.

The four categories of approaches for choosing a subset of features are filter, wrapper, hybrid, and embedded. Regardless of the classification algorithm employed, the filter approach measures different combinations of features and gives them a meaningful score [48]. Two typical measuring criteria in this technique are the correlation criterion and mutual information. The wrapper technique employs an iterative process to assess the usefulness of the subset of features using an assessment criterion that is dependent on the algorithm. Wrapper type is hence the feature selection methodology applied in this study. Three fundamental components make up the feature selection problem: the search method, the subset evaluation, and the stopping condition. Typically, the feature selection procedure is carried out as depicted in Fig. 2.

The feature selection problem is solved using the grasshopper evolutionary method. One of the greatest groups of living things is the grasshopper. The locusts' sluggish, baby-like movements during the larval stage are their primary distinguishing feature. On the other hand, these groups of more adult grasshoppers also share the fundamental trait of long-term and abrupt movement. The search procedure in this algorithm is split into two phases: exploration and exploitation. While during exploitation they prefer to move locally, search agents are pushed to move quickly during exploration [49,50]. Along with target search, locusts intuitively carry out these two tasks. Equation (2) describes the mathematical model used to simulate the behavior of locusts in groups:

(2) $$Y_{i}=C_{i}+H_{i}+B_{i}$$

In the aforementioned connection, *Y*_*i*_ denotes the positional value of the *i-th* propeller, whereas Si represents the social interactions encompassing both attractive and repulsive forces. Additionally, *H*_*i*_ represents the gravity force acting on the *i-th* propeller, whereas *B*_*i*_ denotes the horizontal force exerted by the wind [51]. The parameter *C*, denoted as a function, serves to quantify the magnitude of social forces and is mathematically described by relation (3).

(3) $$s\left(r\right)=fe^{\frac{-r}{l}}-\;e^{-r}$$

**Fig. 3:**
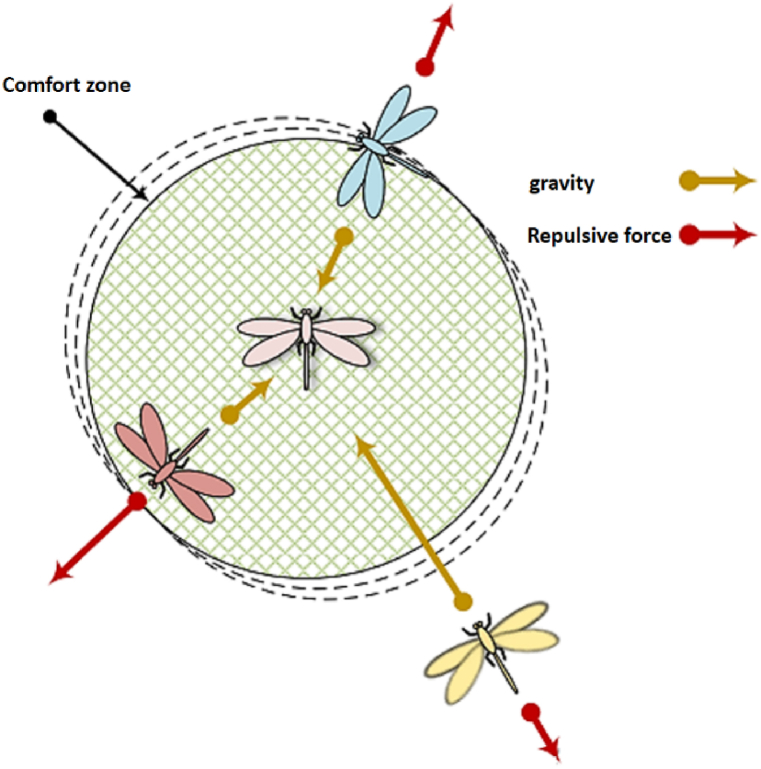
The portrayal of interlocustal dynamics within a collective.

In the aforementioned connection, the variable *f* represents the magnitude of the attractive force, while the variable l denotes the characteristic length scale associated with that attraction. Fig. 3 illustrates the method of visually representing the propellers and the resultant forces acting upon them [52]. The comfort zone can be defined as a state in which there is an absence of both attractive and repulsive forces.

The conventional propeller optimization approach involves assigning varying values to the propeller vector, so establishing and quantifying attraction and repulsion operators inside a continuous space. However, this model may not perform well when it comes to feature selection concerns [53,54]. Consequently, the investigation will employ the binary rendition of the grasshopper algorithm. The steps outlining the suggested strategy for picking appropriate features from the dataset are as follows: The process of establishing the initial settings for the Locust algorithm is of utmost importance. Prior to implementing the proposed way of utilizing the Locust algorithm for feature selection in the dataset, it is important to establish the basic parameters of this approach. Within this context, it is vital to consider many parameters, including the quantity of locusts, the frequency of repeats, the magnitude of characteristics, and the establishment of coefficients. In the suggested grasshopper evolutionary approach, the initial stage involves the creation of a random set [55]. The procedure of generating the random set involves representing the features in the selected subset with the value 1, while the characteristics not included in the selected set are represented with the value 0. Equation (4) shows this process.

(4) *Population= Random Generator (1.. N)*

where *N* represents the quantity of features that have been selected in the preceding stage. The subsequent stage involves the determination of the appropriateness of the chosen set through the use of the suggested fitting function [56]. The fitting function is calculated using formula (5).

(5) $$Fitness=\frac{Acc\left(D\right)}{\left|R\right|}$$

The term *Acc(D)* denotes the degree of accuracy in classifying a data set using a subset of created features. Additionally, *R* represents the count of members inside the subset of selected features. The formula for population fitness incorporates two parameters, namely accuracy and number of characteristics. Indeed, a higher level of accuracy in classification can be achieved by utilizing these extracted characteristics and minimizing the amount of selected features. This leads to an improved fit and better alignment with the target population [57,58]. At this stage, the classification process involves partitioning the dataset into two subsets: 70 % for training purposes and 30 % for testing purposes.

To initiate modifications for the purpose of generating a novel population, the parameters *a* and *a2* will be computed based on equations (6), (7) respectively.

(6) $$a=2-t*\left(\frac{2}{Max_{iter}}\right)$$

(7) $$a2=-1+t*\left(\frac{-1}{Max_{iter}}\right)$$

The parameter *t* denotes the loop index, whereas the variable *Max*_*iter*_ represents the upper limit for the number of iterations associated with the grasshopper mutation process. In the subsequent stage, the parameters A and C will be computed as per the relations (8):

(8) $$\begin{array}{c} A=2*a*r1-a\\ C=2*r2 \end{array}$$

In equation (8), the values of *r*_*1*_ and *r*_*2*_ will be produced randomly within the interval of 0 and 1. Additionally, the parameter denoted as "l" will be determined through the computation of equation (9).

(9) l=(a2−1)*rand()+1

Upon performing the aforementioned calculations, the resultant value of the new answer shall be determined in the subsequent manner:

$$New=Mutation\left(\dim,Max_{iter},Current,subit\right)$$

In the aforementioned formula, the variable "dim" represents the numerical quantity denoting the number of variables [59]. The variable "*Max*_*iter*_" denotes the upper limit of iterations, while "*Current*" represents the current solution. Additionally, "*subit*" corresponds to the loop index. The Mutation function is defined as (10):

r=rand(1..dim)

(10) New(r)=rand(sum(r(:)),1)>0.5

**Fig. 4:**
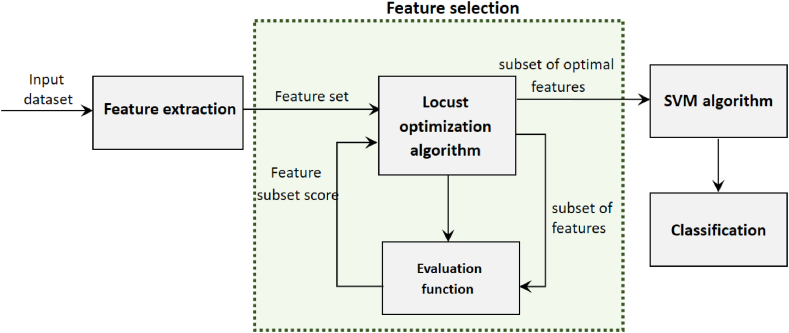
Feature selection system of the proposed method.

According to the explanations, Fig. 4 shows the process of this feature selection algorithm.

As depicted in Fig. 4, the dataset is initially fed into the proposed system. Subsequently, the aforementioned data set is inputted into the grasshopper evolutionary algorithm. The locust algorithm involves the initial selection of a subset from a given dataset. Subsequently, the classification operation is executed on this subset, and the resulting score is delivered to the locust algorithm as its fitness measure [60]. The process continues until a specific iteration is attained, at which point the subset with the highest fitness score is identified and displayed as having the chosen characteristics. Additionally, it is noteworthy to note that the accuracy efficiency criterion was employed in order to compute the fitness value. Indeed, at each stage, the fitness of the dataset is evaluated based on the classification accuracy achieved using the chosen subset.

### 3.4 Process of constructing a support vector machine utilizing the provided dataset

**Fig. 5:**
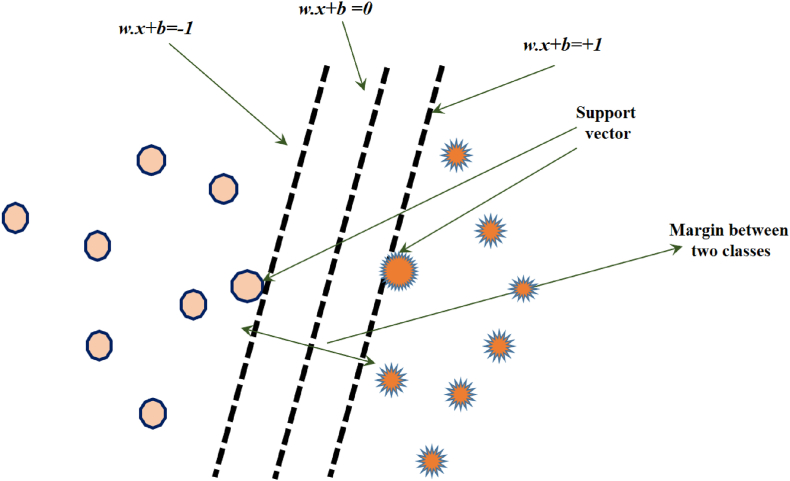
The optimal linear border is determined in the scenario where two classes are entirely segregated from one another.

In this stage, a support vector machine (SVM) is constructed for the purpose of diagnosing cardiac disease. The support vector machine algorithm is a non-parametric supervised statistics method. The primary characteristic of this approach is in its notable capacity to utilize a less number of educational samples while attaining superior accuracy in comparison to alternative categorization approaches. Fig. 5 displays two distinct classes together with their respective support vectors [61,62]. The assumption is made that the dataset comprises two distinct classes, each containing a total of xi training points, denoted by *i=1, …,L*. Each training point *x*_*i*_ is represented as a vector. The two classes are denoted as *y*_*i*_ *= ±1*. Given our objective of categorizing the data into two distinct groups, namely cardiac patients and healthy individuals, it is imperative to treat these classes as entirely independent from one another. To determine the decision boundary between these two different classes, we employ the optimal margin approach. This method involves the calculation of the linear boundary that separates two classes.

• In order to ensure proper classification, it is recommended that all samples belonging to the +1 class be positioned on one side of the border, while all samples belonging to the −1 class be positioned on the other side of the border.
• The optimal choice boundary should be determined in a way that maximizes the perpendicular distance between the nearest educational samples from each class.

In a broad context, a linear decision boundary can be expressed as equation (11).

(11) *w.x + b = 0*

In the aforementioned connection, *x* represents a point situated on the decision boundary, whereas w denotes an n-dimensional vector positioned on the decision border [63,64]. The variable *b* denotes the distance between the origin and the decision boundary, whereas the expression *w.x* indicates the inner product of two vectors, *w* and *x*. The uniqueness of the values of *b* and *w* is defined by applying the following requirements, as equality is preserved by multiplying a constant on both sides of the aforementioned connection. equations (12), (13) show this trend.

(12) *If x*_*i*_*is a support vector y*_*i*_*(w.X*_*1*_*+ b) =1*

(13) *If x*_*i*_*is not a support vector y*_*i*_*(w.X*_*1*_*+ b) >1*

The initial stage in determining the appropriate decision-making boundary entails identifying the nearest instances within the educational dataset that represent two distinct classes. In the subsequent stage, the measurement of the spatial separation between these sites is computed along a vector that is orthogonal to the boundaries that entirely segregate the two categories. The decision border that maximizes the margin is referred to as the ideal decision-making boundary.

#### 3.4.1 Process of forming the separating supersurface

**Fig. 6:**
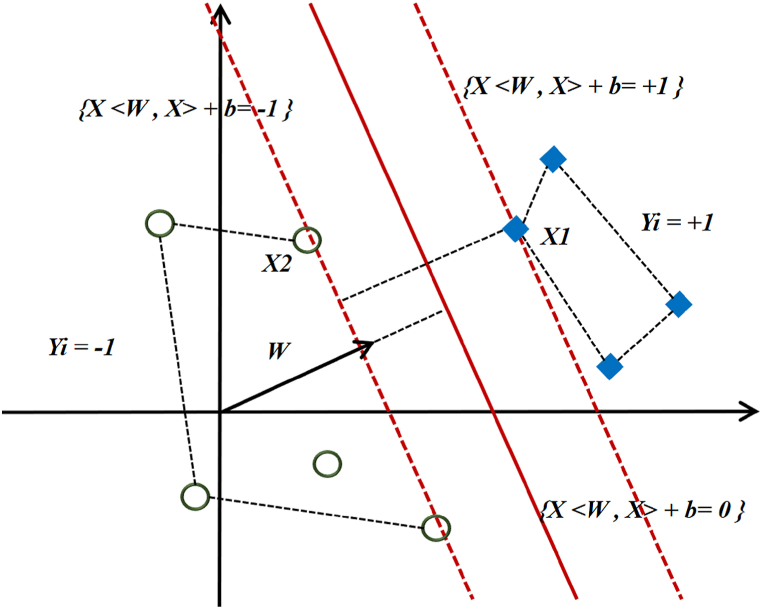
Creating a two-dimensional hypersurface to partition off data layers.

In this section, we want to provide a comprehensive explanation of the procedure for constructing the separating hypersurface using an illustrative case. Fig. 6 illustrates a comprehensive depiction of the formation process of the separation hypersurface by the support vector machine.

Firstly, it is important to investigate the construction of a convex hull that encompasses the set of points belonging to each individual class. Fig. 6 depicts the delineation of a convex hull encompassing the data points associated with class −1 and class +1. Line *P* represents the shortest distance between two convex shells. The separating hypersurface, denoted as *h*, is a linear entity that divides P into two equal parts, bisecting it at the center and being orthogonal to it. The variable b represents the width from the origin for the hypersurface that possesses the maximum separation border. When the variable b is excluded, the resulting solution set consists solely of hypersurfaces that intersect the origin [8,65]. The vertical displacement of the hypersurface from the origin can be determined by dividing the magnitude of the parameter *b* by the length *w*. The primary objective is to select an appropriate delimiter. The term refers to the partition that exhibits the maximum spatial separation from adjacent spots on both levels. Indeed, this solution exhibits the utmost adjacency with the points of two distinct levels and can be enclosed by two parallel hypersurfaces that intersect at least one point from each of the aforementioned levels. The aforementioned vectors are commonly referred to as support vectors. As seen in equations (14), (15), this academic research is related to the construction of a separating hypersurface inside a two-dimensional spatial context, specifically between two separate layers of data.

(14) w.x–b=1

(15) w.x–b=−1

One important observation is that in the case of linearly separable training data, it is possible to choose two boundary hypersurfaces so that there is no data lying between them. By doing so, it becomes feasible to maximize the distance between these two parallel hypersurfaces. By utilizing geometric theorems, it can be determined that the distance between the two hypersurfaces is equal to 2/|w|. Consequently, the value of |w| that minimizes the distance is sought. In order to ensure the exclusion of data points located within the border area, a mathematical constraint is incorporated into the formal definition. By applying restrictions based on relations (16) and (17) for each value of *i*, it can be ensured that no point is located on the border.

(16) $$\begin{array}{c} \text{For}\;\text{the}\;\text{first}\;\text{category}\;\text{data}\\ w.\;x_{i}\;–\;b\geq 1 \end{array}$$

$$\begin{array}{c} \text{For}\;\text{the}\;\text{second}\;\text{category}\;\text{data}\\ w.\;x_{i}\;–\;b\leq -1 \end{array}\;\left(17\right)$$

The aforementioned constraint can be mathematically represented by equation (18).

(18) $$c_{i}\left(w.\;x_{i}\;-\;b\right)\leq 1,1\leq i\leq n$$

In this stage, the output obtained from the feature extraction section is fed into the support vector machine algorithm. The learning process and subsequent classification of heart disease diagnosis are then carried out based on these primary characteristics, which are devoid of any outliers. The process of identifying and selecting the primary characteristics is a fundamental activity that enhances the quality of data. Enhancing the performance of the support vector machine algorithm for the diagnosis and classification of heart disease can be achieved through the acquisition of knowledge pertaining to the primary characteristics offered by the grasshopper optimization algorithm. In methodologies that do not possess this capability, the learning algorithm is compelled to utilize features that do not possess a distinct association with heart disease. This form of learning can be characterized as learning with outlier data, which ultimately exerts a detrimental impact on the classification answer. Additionally, the employed kernel function is derived from the radial basis function (RBF). The Radial Basis Function (RBF) is widely recognized as a prominent kernel function employed in diverse kernel learning techniques. Specifically, it is commonly utilized in support vector machine classification tasks.

### 3.5 Segmentation of training and test data

The next step is to extract a suitable division from the needed data that has the least error and the highest accuracy after the support vector machine of the proposed system has been established. We will experience a condition known as over-training of the model, which creates mistakes as a result of the presence of exceptional occurrences in the training data, if the training of the system increases, i.e., if the data that is used for training and developing the model is a big percentage [66]. slow If the test set is chosen manually, it should be made to ensure that every class category found in the training data is represented in the test data.

### 3.6 Evaluation criteria

#### 3.6.1 Mean squared error MAE

According to relations (19) and (20), this parameter denotes the typical discrepancy between the actual value and the expected value:

(19) $$MAE=\frac{\sum_{i=1}^{n}\left|{value}_{actual}-{value}_{predicted}\right|}{n}$$

*value*_*actual*_ in the formula above represents the estimated value of heart disease, whereas *value*_*predicted*_ represents the actual value of heart disease diagnosis in the data set.

#### 3.6.2 Root mean square error

This parameter, which is defined as the relationship shown below, also refers to the difference between the actual value and the expected value:

(20) $$RMSE=\sqrt{\frac{\sum_{i=1}^{n}{\left({value}_{actual}-{value}_{predicted}\right)}^{2}}{n}}$$

#### 3.6.3 Accuracy

One of the most crucial criteria in current algorithms for disease prediction systems is accuracy, which will be calculated using equation (21):

(21) $$Accuracy=1-(\frac{\sum_{i=1}^{n}abs\left({value}_{actual}-{value}_{predicted}\right)}{n}$$

In fact, the accuracy requirement will be determined by first calculating the average error of the predictions made by the disease prediction system, and then subtracting that number from one.

## 4 Implementation system

A system with a 6.53 GHz CPU, 6 GB of RAM, and Windows 10 64-bit operating system has been used to perform all of the computations for the suggested technique. Additionally, the MATLAB programming language and environment of version 2022b have been used to accomplish the suggested method. It is also important to note that the Veka software's algorithms will be utilized to compare the outcomes of the suggested approach with those of other ways.

### 4.1 Evaluation and simulation

The preprocessing of the data set entered into the MATLAB programming environment is the first step of the suggested procedure. Various approaches to eliminate duplicate values for each characteristic were described in the proposed technique chapter. The duplicate values in the data set and how to eliminate them will be examined in Table 4 in accordance with the justifications provided for these features, and the outcomes will also be examined.

**Table 4:** Statistics-based standards for data set properties.

Row	Feature	Maximum	Minimum	Mean	Std.Dev
1	Age	77	29	54.411	9.04
2	Sex	1	0	0.679	0.468
3	CP	4	1	3.166	0.954
4	Trestbps	200	94	131.646	17.612
5	Chol	564	126	246.738	51.857
6	Fbs	1	0	0.146	0.353
7	Restecg	2	0	0.987	0.995
8	Thalach	202	71	149.606	22.913
9	Exang	1	0	0.328	0.47
10	OldPeak	6.2	0	1.035	1.161
11	Slope	3	1	1.596	0.612
12	Ca	3	0	0.674	0.938
13	Thal	7	3	4.73	1.942

The superfluous fields should be deleted from the set using the pre-processing operation in the following step once the statistical criteria of the set of features have been checked. In other words, it is required to eliminate these duplicate characteristics by employing techniques since some fields in the data set for implementing the proposed method may be unknown. We can mention the average method among records that are similar and the technique of the most likely value among records that are similar as examples of these methods. The data set underwent pre-processing operations using the MATLAB programming language, and the outcomes were examined. Table 5 accurately depicts this operation.

**Table 5:** Operations on the dataset before preprocessing.

Row	Feature	Missing	Distinct	Unique	Missing After Preprocess
1	Age	1 %(4)	41	1 %(4)	0
2	Sex	2 %(8)	2	2 %(8)	0
3	CP	1 %(4)	4	2 %(8)	0
4	Trestbps	0 %(0)	50	6 %(17)	0
5	Chol	1 %(4)	152	20 %(61)	0
6	Fbs	2 %(8)	2	0 %(0)	0
7	Restecg	1 %(0)	3	0 %(0)	0
8	Thalach	0 %(0)	91	9 %(28)	0
9	Exang	1 %(4)	2	0 %(0)	0
10	OldPeak	1 %(4)	40	4 %(11)	0
11	Slope	0 %(0)	3	0 %(0)	0
12	Ca	1 %(4)	4	1 %(4)	0
13	Thal	1 %(2)	3	0 %(0)	0

In fact, the redundant data will be eliminated with the pre-processing operation carried out on the aforementioned data set, leaving the balanced data set prepared to use the suggested way. The proposed method then moves on to the stage of extracting important features from the dataset in order to speed up dataset classification. The third chapter's grasshopper evolutionary algorithm is applied in this process. Table 6 provides a list of the input parameters for the grasshopper evolutionary algorithm used to extract features from the data set.

**Table 6:** Grasshopper evolutionary feature extraction algorithm's input parameters.

parameter	value
Population size	50
Probability of intersection	0.5
The amount of initial weight	0.35
Amount of final weight	0.95
The number of repetitions	100

Additionally, the proposed grasshopper evolutionary technique defines the fitness function for carrying out the feature selection procedure as equation (22):

(22) $$Fitness=\frac{\sum_{i=1}^{n}abs\left(Actual\;Value-Predicted\;Value\right)}{n}$$

where n is the number of records in the data set, original value is the value as it appears in the data set, and predicted value is the value that results from using this feature set with the locust evolutionary algorithm. For instance, suppose the suggested technique chose the following arbitrary subset:

Feature Subset = {3,4,5,9}

The amount of error introduced by using this subset with the support vector machine is then calculated using the suggested fitness function, as shown below:

Fitness{3,4,5,9} = 0.294372.

The best subset, which is the subset with the least amount of error, will be chosen as the optimal subset after completing numerous iterations on the data set to produce several subsets with the degree of fitness. The results of applying the grasshopper evolutionary algorithm to the set to extract the characteristics after entering the initial settings are shown in Table 7.

**Table 7:** Output results of the grasshopper evolutionary feature extraction algorithm.

parameter	value
Category error	0.3841
Selected collection	{2,3,7,9,11,12,13}

**Fig. 7:**
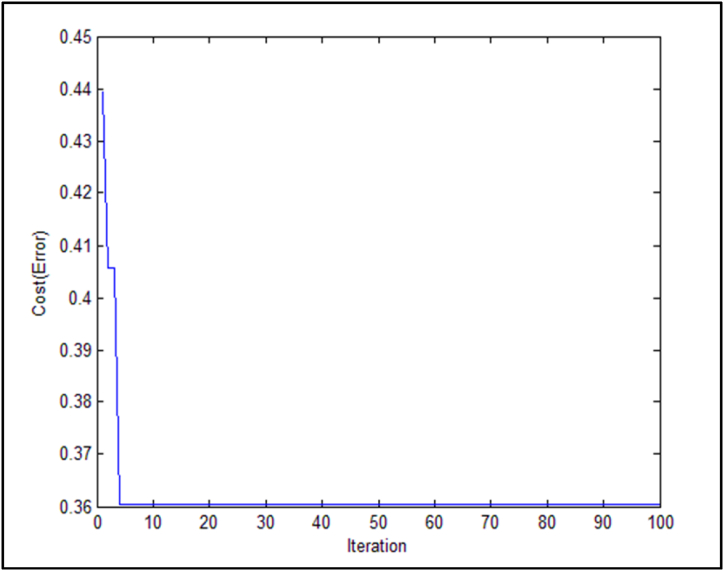
Fitness value for different number of iterations.

Following the application of the evolutionary feature extraction technique of locust, the selected set will consist of seven characteristics and equal the values 2,3,7,9,11,12, and 13, as shown in Table 7. Fig. 7 also displays the categorization error as a function of the number of repetitions.

**Fig. 8:**
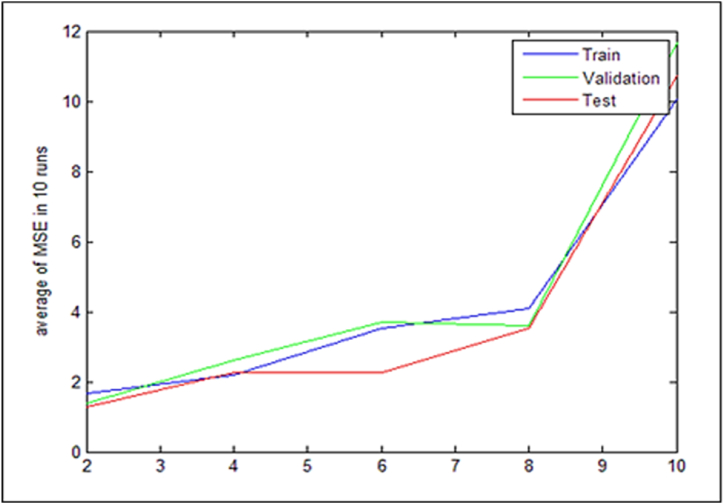
Mean MSE error measure in 10 runs.

The data collection should be categorized using the support vector machine as the following step after employing the grasshopper's evolutionary feature extraction approach. Table 8 lists the fundamental variables. After applying the proposed method to the selected data set, the diagram in Fig. 8 is shown.

**Table 8:** Support vector machine algorithm parameters.

Parameter	Value
Vector number	10
number of repetitions	100
Penalty rate	5/1
Number of executions	5
SVM kernel function type	Radial Basis Function (RBF)
parameter g	32

Fig. 8 displays the average MSE output for three learning, testing, and validation datasets. The support vector machine has been trained using 70 % of the available data. It is important to note that the data set for learning comprises 70 % of the total, testing comprises 15 %, and validation comprises 15 %. The average MSE is low in the first few runs, as seen in Fig. 8, but it rises exponentially in the eighth run. As a result, it was agreed to multiply the quantity of performances by five.

### 4.2 Comparison of the proposed method with other existing methods

We should contrast the suggested method with a few additional methods in the following section of this chapter. He employed the Naive Bayes approach [14], the neural network method [31], and the J48 decision tree method [6] to compare the suggested method to the other 3 methods.

It should be noted that the proposed method, which combines feature extraction based on the support vector machine classification algorithm and the grasshopper optimization algorithm, has been compared with the aforementioned classification methods in this evaluation. The intention is to examine how the feature extraction based on the locust mutation algorithm might enhance the capability of the support vector machine algorithm since the aforementioned algorithms lack the part based on the grasshopper mutation optimization algorithm. We put these three approaches into practice, feed the suggested method's input data set to these algorithms, and then examine the outcomes to compare it to existing methods. The evaluation step, one of the most crucial phases of the classification process, comes after the node classification stage. because it evaluates the categorization model. The label that the classification model gave the new node should be compared to the actual categorization value in order to evaluate. Table 9 displays the occurrence of various node states based on the actual value of combining dataset records for grouping with TP, FP, TN, and FN values.

**Table 9:** Different states for nodes in the network.

Estimated		
	Unauthorized	Allowed
**Allowed**	FN	TP
**Unauthorized**	TN	FP

*TP:* It displays the number of instances in the dataset that have been identified as having heart disease and that have been confirmed as such using the suggested approach.

*FP:* The number of cases that the proposed technique classifies as heart disease yet are classed as healthy in the dataset is represented by the abbreviation FP.

*TN:* is the number of instances in the sample that were identified as having heart disease but were later determined to be healthy.

*FN:* It shows the proportion of instances in the dataset that are labeled as healthy and that were also identified as such by the suggested technique.

Various evaluation criteria have been offered in accordance with the parameters in Table 8, among the most significant of which are the criteria for correctness, accuracy, recall, and criterion F1 stated that these criteria were used to evaluate the built-in models in this study.

The accuracy criteria is the most crucial factor in assessing how effective a classification system is. This metric determines how accurate a category is on the whole. This metric shows the proportion of the full data set that has been accurately categorized. Equation (23) demonstrates how to determine the right criterion.

(23) $$Accuracy=\frac{TP+TN}{TP+FP+FN+TN}$$

The most crucial values that should be maximized to enhance classification effectiveness are TN and TP. The final model will gravitate toward the category with the most samples because there may not be an even distribution of samples across the several categories in node classification issues; instead, one category may have significantly more samples than the other. As a result, the category with a small number of samples will not significantly affect whether performance improves or not. It is clear that the accuracy criterion is inappropriate for the data set's uneven categories and disparate sample sizes. From equation (24), the classification error criteria is also deduced. The accuracy criterion and this relationship are completely at odds with each other. The best efficiency is represented by the lowest value, zero, and the worst efficiency is represented by the highest value, one.

(24) $$ER=\frac{FN+FP}{TP+FP+FN+TN}=1-\text{Accuracy}$$

The accuracy metric displays the proportion of nodes inside the network that the classifier properly assigned to a given category. The relationship (25) has demonstrated this requirement.

(25) $$Precision=\frac{\text{TP}}{\text{TP}+{FP}_{i}}$$

The proportion of nodes correctly classified among all nodes belonging to a category is the criterion for calling the vote in that category. Equation (26), which demonstrates the call criterion calculation process.

(26) $$Recall=\frac{\text{TP}}{\text{TP}+\text{FN}}$$

When no specific weight can be given to any of the two precision and recall criteria individually, the F1 criterion is created by combining the two criteria. This criterion is calculated using Equation (27).

(27) $$F1=\frac{2*\text{Precision}*\text{Recall}}{\text{Precision}+\text{Recall}}$$

**Fig. 9:**
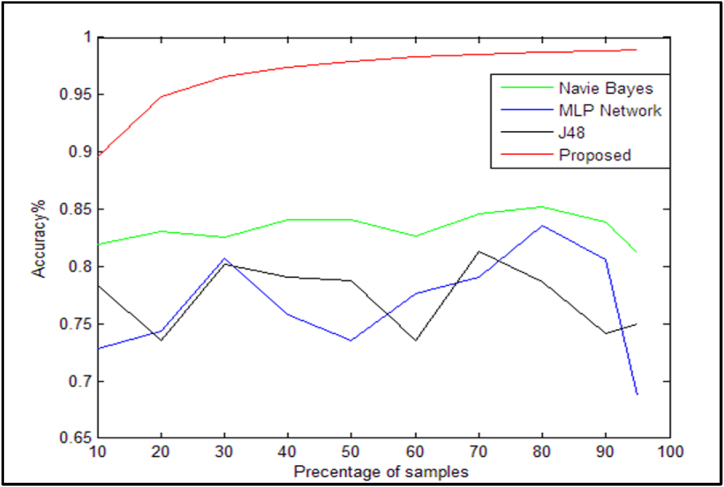
Comparison of the accuracy criteria of the methods presented in Table 10.

The approaches in this section suggest that the output criteria of the suggested method be compared to the algorithms described above as a first step. Since the Naive-Bayesian, neural network, and J48 decision tree methods are unable to extract the primary feature, they execute learning on all sets of data; nonetheless, the vector machine algorithm is utilized in the suggested solution. As a result of the addition of the feature extraction support via the employment of the locust mutation optimization algorithm, the SVM learning process is now limited to the primary features collected from the locust mutation algorithm's output. As will be demonstrated in the following, this capability It has improved the proposed solution's effectiveness and precision. The suggested approach and the algorithms described above are compared using the accuracy criterion as the first criterion in Table 10. Additionally, Fig. 9 more clearly depicts the comparison chart of accuracy standards for the techniques listed in Table 10.

**Table 10:** Accuracy criteria of the proposed method with other presented methods.

Sample/Algorithm	Naïve Bayes [14]	neural network [31]	J48 decision tree [6]	Proposed method
10 %(30)	0.819853	0.727941	0.783088	0.896
20 %(60)	0.830579	0.743802	0.735537	0.948
30 %(90)	0.825472	0.806604	0.801887	0.9653
40 %(120)	0.840659	0.758242	0.791209	0.974
50 %(150)	0.84106	0.735099	0.788079	0.9792
60 %(180)	0.826446	0.77686	0.735537	0.9827
70 %(210)	0.846154	0.791209	0.813187	0.9851
80 %(240)	0.852459	0.836066	0.786885	0.987
90 %(270)	0.83871	0.806452	0.741935	0.9884
95 %(290)	0.8125	0.6875	0.75	0.9892

The proposed method, which uses the locust evolutionary algorithm to increase the accuracy of the support vector machine algorithm, is more optimized than the other methods that have been presented, such as the Navie Bayes method, neural network, and J48 decision tree, as can be seen from Fig. 9. It is merely an idea. The MAE standards are also shown in Table 11 for various sample percentages. Different percentages of the data set were used in this test. In other words, the solutions have been assessed based on the data collected, which ranged from 10 % of the operation to 95 %.

**Table 11:** Comparison of the MAE error measure of the proposed method with existing methods.

Sample/Algorithm	Naïve Bayes [14]	neural network [31]	J48 decision tree [6]	Proposed method
10 %(30)	0.2511	0.3082	0.2983	0.0263
20 %(60)	0.2024	0.2536	0.3128	0.0396
30 %(90)	0.1998	0.2379	0.2487	0.0317
40 %(120)	0.2003	0.2454	0.2611	0.0249
50 %(150)	0.2001	0.266	0.2785	0.0256
60 %(180)	0.2163	0.2508	0.3176	0.0286
70 %(210)	0.2101	0.2274	0.2617	0.0284
80 %(240)	0.1973	0.1946	0.281	0.0290
90 %(270)	0.2477	0.2225	0.2971	0.0295
95 %(290)	0.3029	0.3181	0.2911	0.0295

**Fig. 10:**
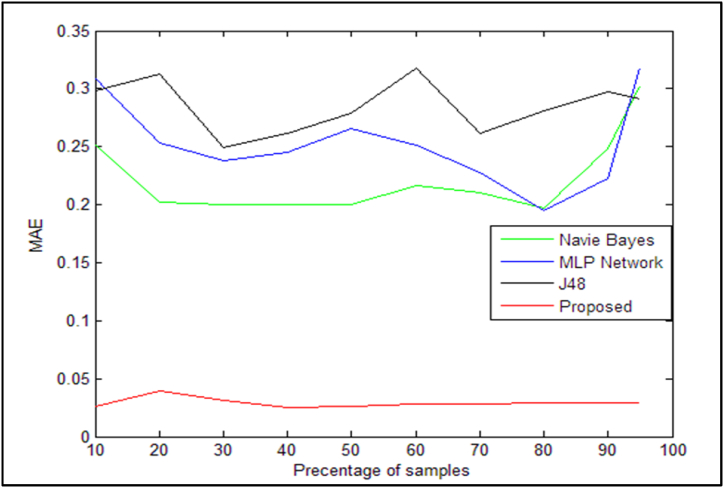
Comparison of the MAE criterion for the methods presented in Table 1.

Additionally, Fig. 10 provides a clearer comparison of the average absolute error standards for the techniques listed in Table 11.

**Fig. 11:**
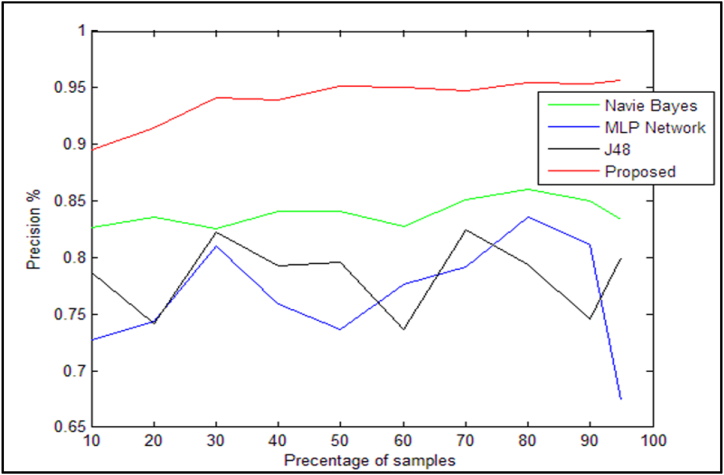
Comparison of accuracy criteria for the methods presented in Table 12.

Fig. 10 makes it evident that the proposed approach has a lower average absolute error threshold than the other methods shown, making it the most ideal option in this regard. The accuracy standards for the suggested method and alternative methods are also contrasted in Table 12. As can be observed, the proposed solution in the sample hut has a higher accuracy than the other solutions that were compared. Taking into account that this approach employs the binary form of the grasshopper leap optimization algorithm. The number of features in the search space will be equal to the vector value of locusts, which is defined in binary form. In other words, the complete collection of data will be the input to the grasshopper leap optimization method. Next, a subset of features will be encoded and shown based on each locust's location in the search space. The key elements that have the greatest influence on the diagnosis of heart disease will be represented by the output result of the solution. Additionally, Fig. 11 makes it easier to compare the accuracy standards for the techniques listed in Table 12.

**Table 12:** Comparison of the accuracy criteria of the proposed method with existing methods.

Sample/Algorithm	Naïve Bayes [14]	neural network [31]	J48 decision tree [6]	Proposed method
10 %(30)	0.8265	0.727	0.7865	0.8947
20 %(60)	0.836	0.744	0.7415	0.9143
30 %(90)	0.8255	0.81	0.822	0.9412
40 %(120)	0.8405	0.7585	0.7925	0.9394
50 %(150)	0.841	0.7365	0.796	0.9518
60 %(180)	0.8275	0.7765	0.7365	0.95
70 %(210)	0.851	0.7915	0.825	0.9478
80 %(240)	0.86	0.836	0.7935	0.9549
90 %(270)	0.8505	0.811	0.746	0.9533
95 %(290)	0.8335	0.6745	0.8	0.9565

**Fig. 12:**
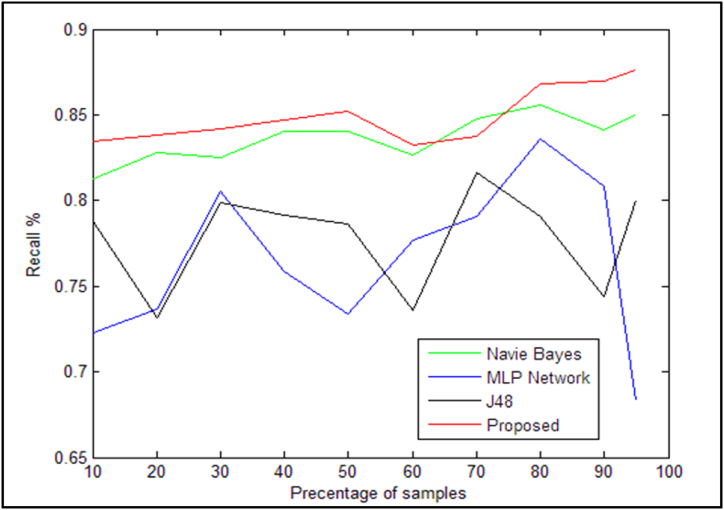
Comparison of recall criteria based on the presented Table 13.

The suggested method's deft criterion is much better than the previous approaches mentioned in Table 13 as illustrated in Fig. 11. The recall requirements for the suggested approach and other methods that are offered are likewise correctly displayed in Table 13. Fig. 12 also shows this comparison more clearly.

**Table 13:** Comparison of the calling criteria of the proposed method with existing methods.

Sample/Algorithm	Naïve Bayes [14]	neural network [31]	J48 decision tree [6]	Proposed method
10 %(30)	0.813	0.723	0.7875	0.8347
20 %(60)	0.828	0.7365	0.7315	0.8379
30 %(90)	0.825	0.805	0.7985	0.8421
40 %(120)	0.8405	0.7585	0.7915	0.8473
50 %(150)	0.8405	0.734	0.7865	0.8519
60 %(180)	0.8265	0.7765	0.736	0.8327
70 %(210)	0.848	0.791	0.816	0.8374
80 %(240)	0.856	0.836	0.7905	0.8681
90 %(270)	0.8415	0.8085	0.744	0.8693
95 %(290)	0.85	0.6835	0.8	0.8762

**Fig. 13:**
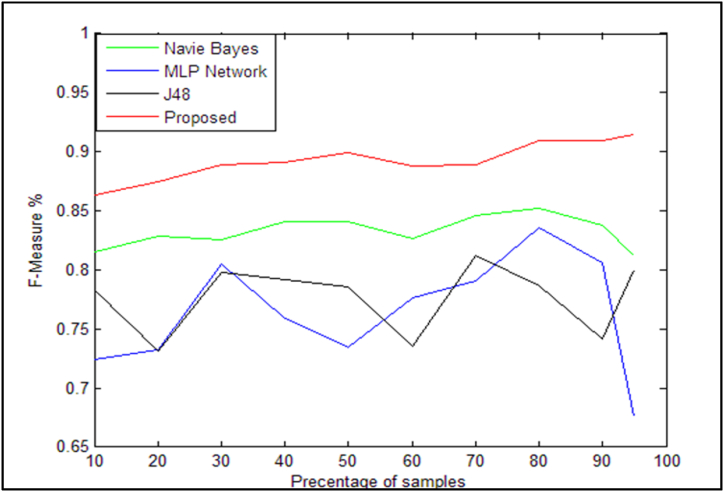
Comparison of the F1 criterion for the algorithms presented in Table 14.

As seen in Fig. 12, the suggested method's calling criterion is significantly better than those of the other algorithms listed in Table 13. The F1 criterion for the suggested method and the other methods discussed is also shown in Table 14. To make the comparison more evident, Fig. 13 is also included.

**Table 14:** F1 criterion for the proposed method compared to existing methods.

Sample/Algorithm	Naïve Bayes [14]	neural network [31]	J48 decision tree [6]	Proposed method
10 %(30)	0.8155	0.7245	0.783	0.8637
20 %(60)	0.8285	0.7325	0.7315	0.8744
30 %(90)	0.8255	0.8055	0.7975	0.8889
40 %(120)	0.841	0.7585	0.7915	0.8910
50 %(150)	0.841	0.734	0.786	0.8991
60 %(180)	0.8265	0.7767	0.7355	0.8875
70 %(210)	0.846	0.791	0.8125	0.8892
80 %(240)	0.852	0.836	0.787	0.9094
90 %(270)	0.838	0.8065	0.7415	0.9094
95 %(290)	0.812	0.676	0.8	0.9146

The proposed method performs better on the F1 criterion than the other methods listed in Table 14 as demonstrated in the diagram of Fig. 13, which compares them.

## 5 Conclusion

Human survival depends on a healthy heart, and if the heart is not operating properly, other body parts, such as the mind and kidneys, may also be impacted. In the entire world, cardiovascular disorders are the leading cause of death. The World Health Organization estimates that cardiac disorders are responsible for 12 million deaths worldwide. Cardiovascular disease is the leading cause of mortality in Iran, and 38 % of Iranians die from heart disease, which is a very high percentage. Experts claim that the emerging topic of data mining is one of the knowledge areas that has recently cemented its place across all disciplines.

Therefore, it is crucial to diagnose this illness without employing any intrusive procedures. Many data have been gathered for this purpose during tests and examinations of cardiac patients, which will be expensive to handle using conventional techniques. By reviewing previous techniques for the diagnosis of cardiac disorders, an attempt has been made in this study to propose a new way in order to increase the accuracy of prediction results.

Based on this, the support vector machine has been applied in this research's proposed strategy to expedite the diagnosis and prognosis of heart illness. The heart disease diagnostic is the output of the proposed support vector machine, which has several inputs and one output. Additionally, features from the data set have been chosen using the grasshopper evolutionary algorithm. Based on the output results attained, the proposed method's efficiency criteria have greatly improved when compared to existing approaches. Therefore, it has been found that the accuracy criteria for the proposed method compared to Neobizin, neural network, and j48 tree methods are 18 %, 30 %, and respectively after implementing the proposed method on the data set and comparing it with other algorithms performed in the field of heart disease diagnosis. 24 % better. In the following, we discuss some limitations of the proposed method in this research:

Algorithms may have sensitive parameters that require fine-tuning. Failure to properly set these parameters can reduce the accuracy and efficiency of the algorithm. In the case of the SVM algorithm, there are limitations related to kernels (such as gamma and C parameters) and limitations related to linear or non-linear kernels, the incorrect selection of which can have a great impact on the performance of the algorithm.

*Limitation in the selection of features:* It is very important to select the appropriate features for use in algorithms. If the appropriate features are not selected or important features are not available in the data, the accuracy and efficiency of the algorithm will decrease.

*Limitation in data and clinical cases:* Limitation in access to complete and comprehensive data related to cardiac patients may reduce the accuracy of disease analysis and prediction. Also, incomplete or incorrect information may have a negative effect on the accuracy of algorithms.

## 6 Future work

The following ideas are recommended for further research based on the findings of this study.

• Employing several evolutionary techniques to enhance the suggested support vector machine: The application of novel evolutionary algorithms, such as the gray wolf algorithm, the weed algorithm, and the evolutionary algorithm of cats, is one area that can be employed to enhance the suggested approach.
• Using different types of data mining to analyze and forecast the data set: Using other categories, such as decision trees, neural networks, etc., can be another way to enhance the suggested approach.
• The selection of features from the data set using evolutionary algorithms: Utilizing additional novel evolutionary techniques, such as the dragonfly algorithm and the evolutionary algorithm of wild cats, among others, is another way to enhance the proposed approach.

## Ethics statement

Ethics committee review and/or approval was not required for this study, as no animal or human-based experiments/case studies were used.

## Data availability statement

Data will be made available on request.

## Funding

This research received no specific grant from any funding agency in the public, commercial, or not-for-profit sectors.

## CRediT authorship contribution statement

**Wei Zhou:** Funding acquisition, Conceptualization. **Hongbo Liu:** Validation, Supervision. **Rui Zhou:** Writing – original draft, Validation, Supervision, Investigation. **Jiafu Li:** Funding acquisition, Formal analysis, Data curation. **Sina Ahmadi:** Writing – review & editing, Supervision, Software, Project administration.

## Declaration of competing interest

The authors declare that they have no known competing financial interests or personal relationships that could have appeared to influence the work reported in this paper.
